# Injectable crosslinked HA hydrogel: a promising carrier for cell transplantation to treat stable vitiligo

**DOI:** 10.3389/fmed.2025.1583271

**Published:** 2025-05-12

**Authors:** Qianren Zheng, Jie Chen, Yixun Huang, Weikai Chen, Dandan Cheng, Qianqian Jia, Meiqin Zhu, Youguo Liao, Qiulin He, Shunli Wu

**Affiliations:** ^1^Hangzhou Singclean Medical Products Co., Ltd, Hangzhou, China; ^2^Department of Student Affairs, Jiaxing Vocational Technical College, Jiaxing, Zhejiang, China; ^3^Department of Orthopedics, The Second Affiliated Hospital and Yuying Children’s Hospital of Wenzhou Medical University, Wenzhou, Zhejiang, China; ^4^Department of Gynecological Oncology, Wenzhou Central Hospital, Wenzhou, Zhejiang, China; ^5^Department of Burns and Wound Care Center, Second Affiliated Hospital, College of Medicine, Zhejiang University, Hangzhou, China; ^6^Department of Macromolecular Science, Fudan University, Shanghai, China; ^7^College of Marine Life Sciences, Ocean University of China, Qingdao, Shandong, China

**Keywords:** autologous epidermal melanocytes, crosslinked hyaluronic acid hydrogel, cell therapy, stable vitiligo, nude mice

## Abstract

Stable vitiligo significantly impacts patients’ quality of life and presents a considerable challenge to healthcare providers. In recent years, cell therapy has emerged as a promising treatment for stable vitiligo, which is demonstrated encouraging results. Among current cell-based therapies, autologous epidermal cell transplantation is regarded as a safe and cost-effective strategy. However, the therapeutic outcome critically depends on the retention and viability of the transplanted cells at the target site. Therefore, there is an urgent need to develop novel strategies to improve cell retention and maintain cell viability for improving therapeutic efficacy. In this work, a novel cell extraction method was first developed with deal with 2 h at 37°C to obtain epidermal cells while maintaining high cell viability. Subsequently, the crosslinked hyaluronic acid (HA) by BDDE was utilized as 3D scaffold for cell delivery to treat stable vitiligo. By combining the new extraction method with the HA-based hydrogel scaffold, we achieved prolonged cell retention without compromising cell viability. This approach provides a promising, time-saving strategy for treating stable vitiligo using autologous epidermal cells.

## 1 Introduction

Vitiligo is a common clinical skin disease, clinically characterized by the loss and absence of melanocytes, and then forming the white spots on the skin (1). The destroy mechanisms of the melanocytes was study in vitiligo disease, and these involve the genetic, oxidative stress, autoimmune, hurt, even the environmental effects (2). Thus, vitiligo treatment strategies often include repair, replenishment, and regeneration of melanocytes (3, 4). The global prevalence of the disease ranges from 0.5 to 2.0% and is increasing annually (5). Thus, there is an excruciating demand for effective, appropriate and safe strategy to treat vitiligo.

Up to now, clinically treatments of vitiligo is including drug therapy (e.g., Janus Kinase Inhibitors), narrow-band phototherapy, epidermal transplantation and cell transplantation, typically (6). However, the treatment effect of drug and narrow-band phototherapy therapy is not significant, and the treatment cycle is longer (7, 8). The epidermal transplantation for vitiligo will cause a large area of skin damage and more scars, which is not suitable for scar constitution (9, 10). The cell transplantation has been catching the attention of physicians and scientists, due to its promising efficacy in treating stable vitiligo (11). For cell suspension injection, the healing outcome was greatly affected and reduced, because of the loss of the implanted cells (11, 12). Thus, the retention and viability of the transplanted cells at the vitiligo sites determine the healing outcome (13). To solve the above problems, biomaterial-based cell delivery systems have been introduced in cell transplantation therapy techniques.

Recently, various of biomaterials were applied to cell transplantation therapy, due to the good biocompatibility and degradability of biomaterials, maintain cell morphology and promote cell migration in a certain extent, such as collagen, chitosan, and hyaluronic acid hydrogel (14–16). Collagen is mostly used in patch form as a carrier for epidermal cell suspensions (17, 18). It usually involves applying the cell suspension to the post-dermabrasion lesion area and then covering it with a collagen patch to prevent the loss of epidermal cells (19). Similarly, Fan et al. preparate a chitosan membrane with melanocytes multicellular spheroids to transplant cells on the skin. The results indicated that the combination of chitosan-based melanocyte spheroid patch can facilitate melanocyte transplantation in the epidermal ablation model by PUVA-induced sunburn reaction (20). Ghorbani et al. used the autologous non-cultured and trypsinised melanocyte suspension mixed with hyaluronic acid gel to spread over the vitiligo area. The results showed that this is a safe with satisfactory strategy in recalcitrant vitiligo by the autologous non-cultured and trypsinized melanocyte grafting (21). In fact, most of the research on the combination of biomaterials and melanocytes in the vitiligo treatment is applying, covering paste, or applying with cell infiltration. However, there are some drawbacks, such as low cell survival due to lack of oxygen and nutrients. It is necessary to change the covering frequently, etc. Thus, a novel strategy to treat vitiligo is urgently developed.

Hyaluronic acid is one of the main components in the skin extracellular matrix (ECM) (22). Hyaluronic acid produced by micro-organisms fermentation, is superior in bio-safety compared with animal tissue-derived biomaterials (23, 24). Currently, Hyaluronic acid-based biomaterials had been widely used for treating skin diseases (25). For example, Wang et al. developed a novel hydrogel system incorporating olive leaf-derived exosome-like nanovesicles loaded with hyaluronic acid and tannic acid. The results demonstrated that this composite hydrogel effectively mitigated UV-induced skin damage while promoting cutaneous repair and regeneration. Furthermore, RNA sequencing and cluster analysis of predicted miR-168a-5p targets revealed significant downregulation of the NF-κB signaling pathway, which mediates inflammaging responses (26). However, the hyaluronic acid solution could not provide enough support for long-term cell retention due to its fragmented molecular chain and fast biodegradation. Fortunately, cross-linked HA can be used to solve these difficulties. Usually, the BDDE (butylene glycol glycidyl ether), DVS (divinylsulfone), ADH (diacetyl hydrazide), EDC (carbodiimide), GMA (glycidyl methacrylate) and PEG were used to crosslink HA to form stable 3D structure (27–31). But the most important processes are the most mature are BDDE and DVS (32). However, the most important process, the most mature and the most widely commercialized is BDDE (33). Its crosslinking ability is attributed to the reactivity of the epoxy groups at both ends of the molecule. Under alkaline conditions, these epoxy groups preferently react with the most accessible primary alcohols in the hyaluronic acid skeleton to form ether bond connections (34).

**SCHEME 1:**
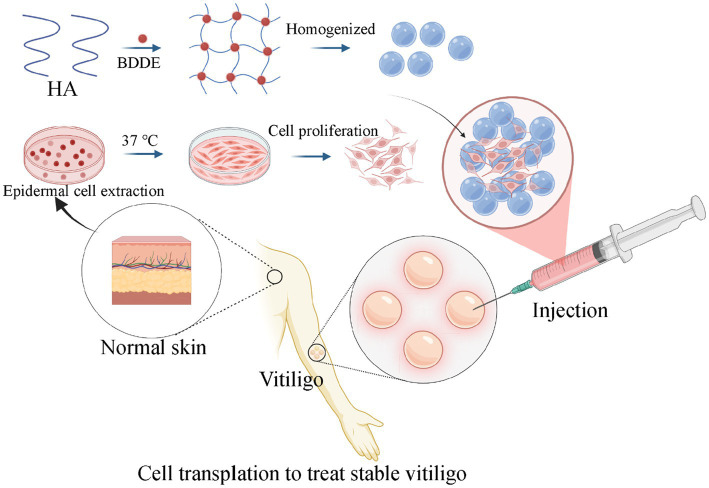
The process of the injectable crosslinked HA hydrogel for cell transplantation to treat stable vitiligo.

Therefore, in this work, crosslinked hyaluronic acid (HA) by BDDE was utilized for cell delivery. Crosslinked HA possesses a three-dimensional structure, which may contribute to slower biodegradation and prolonged retention of transplanted cells. *In situ* injection therapy, the body can provide nutrients and provide a 3D microenvironment for survival, ensuring efficient cell survival. In addition, the introduction of biomaterials can improve cell retention and reduce cell loss. Based on this rationale, an HA-based cell delivery system was developed for the treatment of stable vitiligo (Scheme 1). The first step involved optimizing the extraction method for epidermal cells to ensure a sufficient yield of highly viable cells (~85% viability) within a significantly reduced processing time. Subsequently, an HA-epidermal cell suspension was prepared for *in vivo* cell delivery. The results from animal experiments demonstrated enhanced cell retention without compromising cell viability. Finally, given that HA is derived from a registered medical device, this approach represents a highly efficient and promising method for the clinical treatment of stable vitiligo.

## 2 Materials and methods

### 2.1 Materials

Hyaluronic acid was obtained from A.V.T. The HA molecular mass is 2.5 million. The BDDE was purchased by Sigma. The trypsin–EDTA, alcohol, MTT were buy from Shanghai Beyotime Biotechnology Co. The mouse IgG-PE isotype antibody, rabbit IgG-PE isotype antibody, anti-cytokeratin 14-PE, anti-Pan-cytokeratin-PE, and a-MelanA-PE antibodies were purchased from Abcam.

### 2.2 Crosslinked HA preparation

The 10 g HA powder was dissolved in 100 mL 1% NaOH at room temperature. Then, the BDDE was dropped into the HA solution and mixed for 15 min. After that, the mix solution was bathed at 50°C water for 1 h. After that, the mixture was placed over night at 4°C. Then, the crosslinked HA was dialysis in PBS for 4 days (8,000 DW, change the dialysate twice a day) at room temperature. The crosslinked HA was qualitied to 20 mg/mL PBS smashed by homogenizer with 4,000 rpm. Lastly, after filling, the cross-linked HA gels were sterilized at 121°C for 15 min.

### 2.3 Rheological behavior

To study the rheological behavior of the crosslinked HA hydrogels. The rheological behavior of the crosslinked HA hydrogels was tested by the Austrian Anton Paar Advanced Rotational Rheometer (Physica MCR301, MCR50). The adjustment frequency is constant to 10 Hz, the amplitude range is 0.01%–100%, and the normal force is 5 N.

### 2.4 SEM characterization

First, the crosslinked HA hydrogels were lyophilized by freeze dryer. Then, the conductive Au coating was prepared on the surface. The surface morphologies of the crosslinked HA hydrogels were observed by the scanning electron microscope (SEM, HITACHI, Japan).

### 2.5 Swelling and degradation behavior

The swelling and degradation ratio of the crosslinked HA hydrogel was measured by gravimetric method (35). Al the dry samples (W_0_) were infiltrated into the 10 mL of PBS and shaken (120 rpm, 37°C). Then, the samples were weighed (Ws) after removing surface moisture at 1, 2, 4, 8, 24, and 48 h.

The measure equation of the swelling ratio (Es) as follow:


$$\mathrm{Es}=[(\mathrm{Ws}-W_{0})/W_{0}]\times 100\%(n=5)$$

The dry weight (Wt) of freeze-dried samples was measured at 1, 3, 5, 7, 14, and 28 days to evaluate the degradation ratio (D).

The measure equation of the degradation ratio (D) as follow:


$$D=(W_{0}-W_{t})/\mathrm{W0}\times 100\%(n=5)$$

### 2.6 DLS method measurement settings

1 g crosslinked HA hydrogel in 20 mL ultrapure water was stirred for 15 min. Take an appropriate amount of gel, slowly add it to the detection tank, and DLS measures the particle size.

### 2.7 Extraction of epidermal cells and preparation of suspensions

The human skin sample (biopsy) was washed 2–3 times with saline to remove blood and other contaminants at room temperature. Then, the sample was immersed in 75% ethanol for 1 min. The residual ethanol was removed by saline. The sample (biopsy) was carefully excised subcutaneous tissue. The remaining tissue was cut into small pieces for further processing. All skin samples were by the Second Affiliated Hospital of Zhejiang University and approved by the hospital committee. Skin samples only for scientific researching have been informed to donors and consent obtained.

Following enzymatic treatment, the epidermal tissues were rinsed with saline to remove residual agents. The dermis was mechanically separated from the epidermis by forceps. The epidermis was transferred to a 15 mL centrifuge tube containing an appropriate volume of saline. The epidermis tissue was gently dissociated by pipetting approximately 30 times and then filtered through a 40 μm cell strainer. The filtrate was centrifuged at 300 × g for 5 min at room temperature, and the supernatant was discarded. The cell pellet was resuspended in an appropriate volume of saline for further use.

For the new extraction method (NEM), skin tissues were treated with 0.5% trypsin–EDTA solution and incubated at 37°C for 2 h. For comparison, the traditional extraction method (TEM) was employed, in which skin tissues were treated with 0.25% trypsin–EDTA solution and incubated at 4°C for 16 ± 1 h.

### 2.8 Cell activity assay

Epidermal cells, extracted by both TEM and NEM, were stored at 4°C for varying time intervals. Cell viability was evaluated at 1, 2, and 3 h via the MTT assay (Shanghai Beyotime Biotechnology Co.). Moreover, both TEM and NEM group were co-incubated with cross-linked HA extract (37°C, 3 day) for 24 h.

### 2.9 Cell surface marker assay

Cell samples (1 × 10^6^ cells per sample) from each group were centrifuged at 40 × g for 5 min. The cells were resuspended in 0.2% Triton X-100 solution, lysed for 15 min at room temperature (RT), and centrifuged again at 40 × g for 5 min. The supernatant was discarded, and the cell pellets were washed with PBS. Blocking was performed by serum for 1 h at RT, followed by another centrifugation step at 40 × g for 5 min. The supernatant was removed, and the cell pellets were washed with PBS. Each sample was then resuspended in 100 μL of staining buffer.

For the blank control group, no antibody was added (negative control group). The cytokeratin 14 and Pan-cytokeratin were used to detect the presence of keratinocytes. The a-MelanA was used to detect the presence of melanocytes and outline their distribution. For the isotype control group, mouse IgG-PE isotype antibody (Abcam, ab91357, 20 μL/10^6^ cells) and rabbit IgG-PE isotype antibody (Abcam, ab209478, 0.1 μg/mL and 0.5 μg/mL) were added as single stains. For the experimental groups, the following antibodies were added: anti-cytokeratin 14-PE (Abcam, ab211997, 0.1 μg/mL), anti-Pan-cytokeratin-PE (Abcam, ab52460, 1 μg/mL), and *α*-MelanA-PE (Abcam, ab225499, 0.5 μg/mL). All samples were incubated for 30 min at 4°C in the dark. After incubation, the samples were centrifuged at 40 × g for 5 min. All the cell pellets were washed with PBS. Finally, the cell pellets were resuspended in 500 μL of staining buffer and subjected to flow cytometry for analysis. Gating strategy: (1) FSC-A/SSC-A gate main cell population; (2) Count/PE-A gate PE positive and PE negative groups. According to the above gating strategy, the negative expression regions were determined by Blank and Isotype, and the positive expressions of cytokeratin 14, Pan-cytokeratin and a-MelanA in each sample were analyzed.

### 2.10 Animal experiments

Forty-eight nude mice were randomly divided into four groups, with an equal distribution of males and females: (1) the new technology HA cell suspension group (NEM + HA), (2) the traditional technology HA cell suspension group (TEM + HA), (3) the new technology cell suspension group (NEM), and (4) the traditional technology cell suspension group (TEM). Each group received a subcutaneous injection of 100 μL of cell suspension (3 × 10^7^ cells/mL) into the dorsal region, with the injection site clearly labeled. All animal experiments are carried out by third-party animal testing companies. All animal experimental procedures are approved by the Ethics Committee of Hangzhou Hebei Technology Co., LTD (ethical approval number: HBQR4.05-04).

The inoculation sites were photographed and evaluated for skin discoloration on day 7 post-inoculation. At the endpoint of observation (day 7), animals in each group were euthanized. The skin at the inoculation site was carefully excised using ophthalmic scissors, and the biopsies were fixed and embedded in wax for further analysis.

### 2.11 Masson-Fontana staining

Dewaxing was performed by xylene solution twice (15 min each time). Then, the samples were immersed in Fontana ammonia-silver solution at 56°C for 40 min, followed by six rounds of washing with water (2 min each). Next, the samples were treated with hypo solution for 5 min and rinsed with water for 5 min. Re-staining was performed using neutral red staining solution for 5 min, followed by a 1-min water wash. The tissues were dehydrated through a gradient alcohol series (95, 100%) for 0.5 min each and cleared with xylene twice (2 min each). Finally, the samples were mounted with neutral gum, observed under a microscope, and photographed.

### 2.12 Immunohistochemistry staining

Dewaxing was performed using xylene solution twice, 20 min each time. The samples were then treated with a gradient alcohol series (100, 100, 95, 80%) for 5 min each, followed by three rinses with phosphate-buffered saline (PBS) (3 min each). The samples were incubated in 3% hydrogen peroxide at 37°C for 10 min and rinsed with PBS three times (3 min each). Next, the samples were placed in 0.01 M citrate buffer (pH = 6.0) and boiled for 10 min, followed by natural cooling to room temperature. After cooling, the samples were rinsed with PBS three times (3 min each). Anti-Human Nuclear Antigen antibody (ab191181, Abcam) was applied, and the samples were incubated overnight at 4°C. The samples were then transferred to room temperature (RT) for 30 min and rinsed with PBS. Goat anti-Mouse IgG H&L (ab205719, Abcam) was added, and the samples were incubated at 37°C for 30–40 min, followed by a PBS wash. For visualization, DAB (3,3′-diaminobenzidine) was used for coloration. Finally, hematoxylin was applied for nuclear staining, and the slides were mounted with neutral gum for microscopic examination.

### 2.13 Statistical analysis

The One-way ANOVA way was played to evaluate the statistical significance by the GraphPad Prism 8 software. All the data were analyzed and obtained from over triplicate samples.

## 3 Results

### 3.1 Crosslink HA hydrogel preparation and character

**Figure 1:**
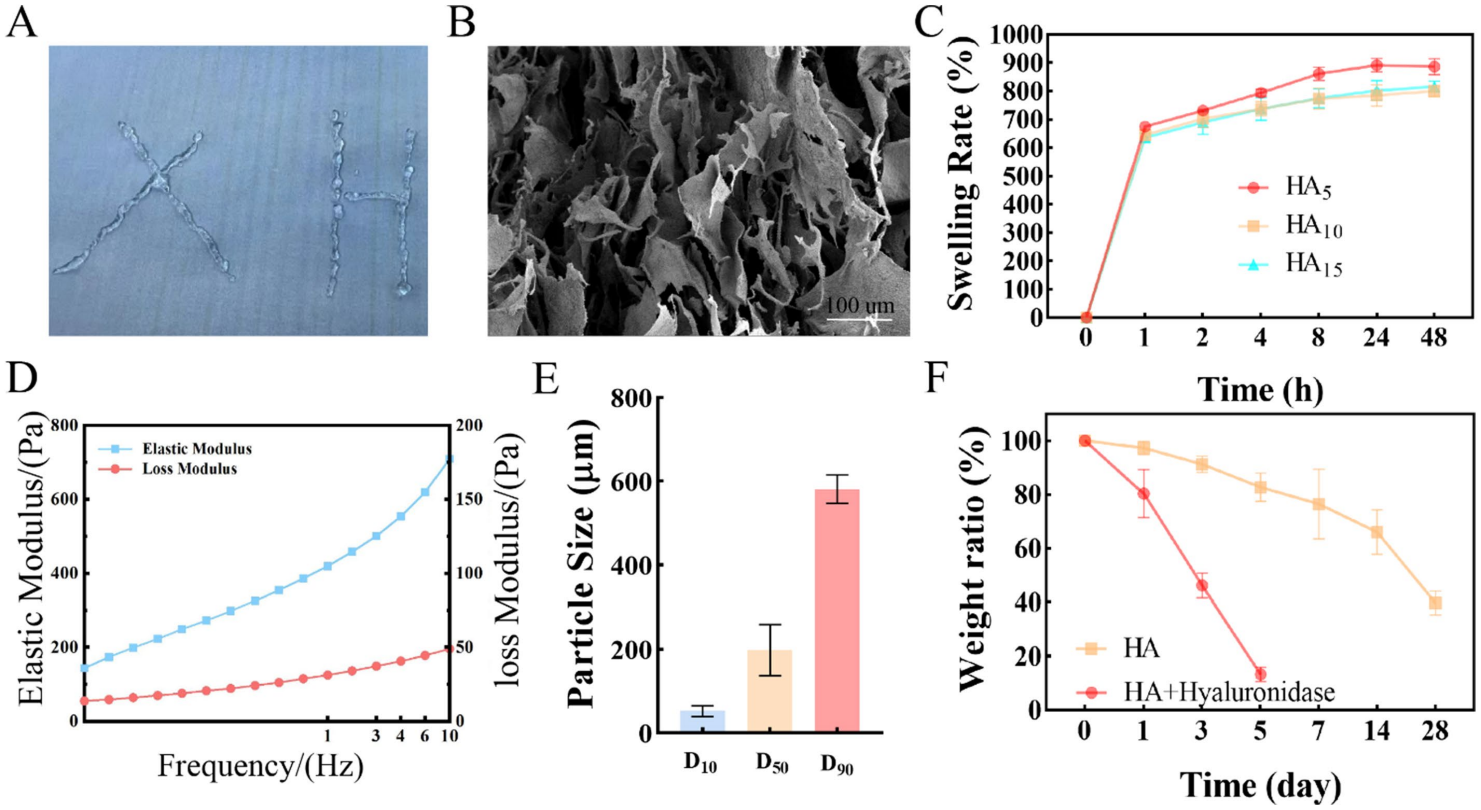
The characteristics of Crosslink HA hydrogel. **(A)** Macroscopic diagram of cross-linked HA. **(B)** SEM characteristic of cross-linked HA. **(C)** The swelling rate characteristics of Crosslink HA hydrogel. **(D)** the Rheological properties of cross-linked HA. **(E)** Cross-linked HA particle size distribution. **(F)** The weight rate characteristics of Crosslink HA hydrogel.

In this work, a type of crosslinked HA hydrogel was fabricated by the BDDE at 4°C overnight. Then, the crosslinked HA hydrogel was smashed by homogenizer. The crosslinked HA product a continuous transparent gel state (Figure 1A). As shown in Figure 1B, the crosslinked HA hydrogel showed a pore structure in solid gel from the SEM. This is because the HA hydrogel particle own high adhesion resulting in the hydrogel particle form a solid gel and with high pore rate. The crosslinked HA hydrogel swelling rate was tested via the weighing method. The results showed that the swelling rate reached a swelling balance PBS at 37°C after 24 h. Interestingly, the swelling rate of the crosslinked HA is decreased with the increase of crosslinker BDDE (Figure 1C). In the same case, the swelling equilibrium of cross-linked HA was 889.2% when the crosslinker was added to 5 ul, and when the crosslinker was added to 10 ul, the swelling balance of cross-linked HA was 815.3%. Importantly, after adding 10 ul and 15 ul crosslinker to HA, the swelling rate is similar (about 816%). This may be due to the fact that the optimal crosslinking ratio has been reached at 10 ul of crosslinker. However, the result (Supplementary Figure S1) showed that the swelling rate of crosslink HA hydrogel in wet condition was 128.2%, which had no significant changes compared to initial wet hydrogel. Thus, it should have no detrimental effects on the patient. Additionally, the rheological behavior of the crosslink HA hydrogel. The results (Figure 1D) indicated that the elastic modulus (G′, 121.4 Pa) is higher than the loss modulus (G″, 405.8 Pa). This is due to the fact that the cross-linked gel is a solid structure. Furthermore, the particle size of crosslinked HA hydrogel was D_10_ = 52.2 ± 3.2 μm, D_50_ = 198.1 ± 7.1 μm, D_90_ = 581.5 ± 5.4 μm (Figure 1E). The highly interconnected and distributed porous structures may provide a large number of adhesion sites and create a favorable microenvironment for cell adhesion and migration. To mimic the microenvironment, the hydrogels were incubated at 37°C in PBS with or without hyaluronidase. After being incubation, the samples were taken out and lyophilized, and then monitored the remaining weight. The HA hydrogel degraded the fastest and decreased to 39.6%% of the original mass after 28 days in PBS. However, while the HA hydrogel with hyaluronidase (150 U), the remaining weight decreased to 13.2%, only in 5 days (Figure 1F).

### 3.2 Epidermal cells extraction and preservation

**Figure 2:**
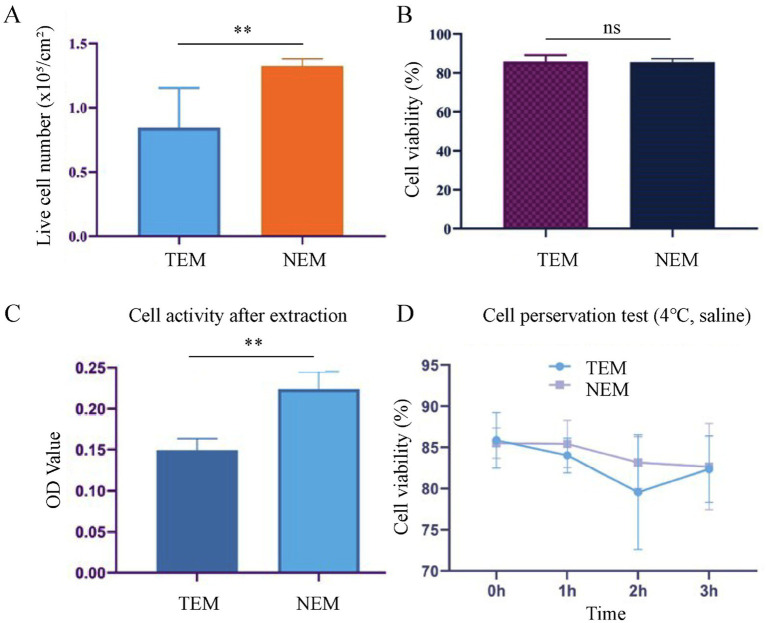
Cell extraction and preservation. **(A)** Live cell number per unit area after extraction. **(B)** Cell viability after extraction. **(C)** Cell activity after extraction, evaluated by MTT assay. **(D)** Cell viability after preserved in saline at 4°C for different time intervals (*n* = 6, mean ± SD) (^**^p < 0.005).

At first, we optimized the protocol for extracting autologous epidermal cells. In addition, we further demonstrated that the extracted cells were epidermal cells and not fibroblasts by comparing the morphology of the extracted cells and fibroblasts (Supplementary Figure S2). We compared the viability and density of cells extracted by two different methods: (1) the TEM, which involved 0.25% trypsin–EDTA solution at 4°C for 16 ± 1 h, and (2) the NEM, which utilized 0.5% trypsin–EDTA solution at 37°C for 2 h. On average, the yielded cell viability is about 85.88% and a cell density is 8.44 × 10^5^/cm^2^ in TEM group (control). the NEM group is achieved a viability of 82.39% and a density of 1.13 × 10^6^/cm^2^ (Figures 2A,B). These results demonstrate that the new extraction technique not only significantly reduces the time required for cell extraction but also maintains high cell density and viability.

Next, the cell proliferation activity of cells, obtained from two extraction methods, was assessed via the MTT assay. After 3 days of culture, the average absorbance value for the experimental group (NEM) was 0.224 ± 0.018, while the control group (TEM) showed an average absorbance value of 0.149 ± 0.013. These results indicate that cells extracted via the NEM exhibited significantly higher proliferation activity compared to those from the control group (Figure 2C).

To establish the optimal timeframe for clinical application, we systematically evaluated the impact of storage duration on cellular viability. Cells isolated by both conventional (TEM) and novel (NEM) extraction methods were suspended in saline and maintained at 4°C for defined intervals (0–3 h). Viability assessment at each time point demonstrated consistent maintenance of cellular integrity, with NEM-isolated cells exhibiting viabilities of 85.52 ± 0.8% (0 h), 85.43 ± 1.2% (1 h), 83.16 ± 1.5% (2 h), and 82.66 ± 1.1% (3 h). TEM-isolated controls showed comparable stability (85.88 ± 0.9%, 84.04 ± 1.3%, 79.57 ± 1.7%, and 82.37 ± 1.4% at respective time points) (Figure 2D). Statistical analysis confirmed no significant viability reduction (*p* > 0.05) throughout the 3-h observation period for either method. These findings indicate a clinically practical ≥3-h window for surgical preparation while maintaining optimal cellular viability. Moreover, after 24 h of co-incubation with the cross-linked HA extract, the cell viability was as high as 95%. This demonstrates that cross-linked HA has a high biosafety (Supplementary Figure S3).

### 3.3 Cell safety

**Figure 3:**
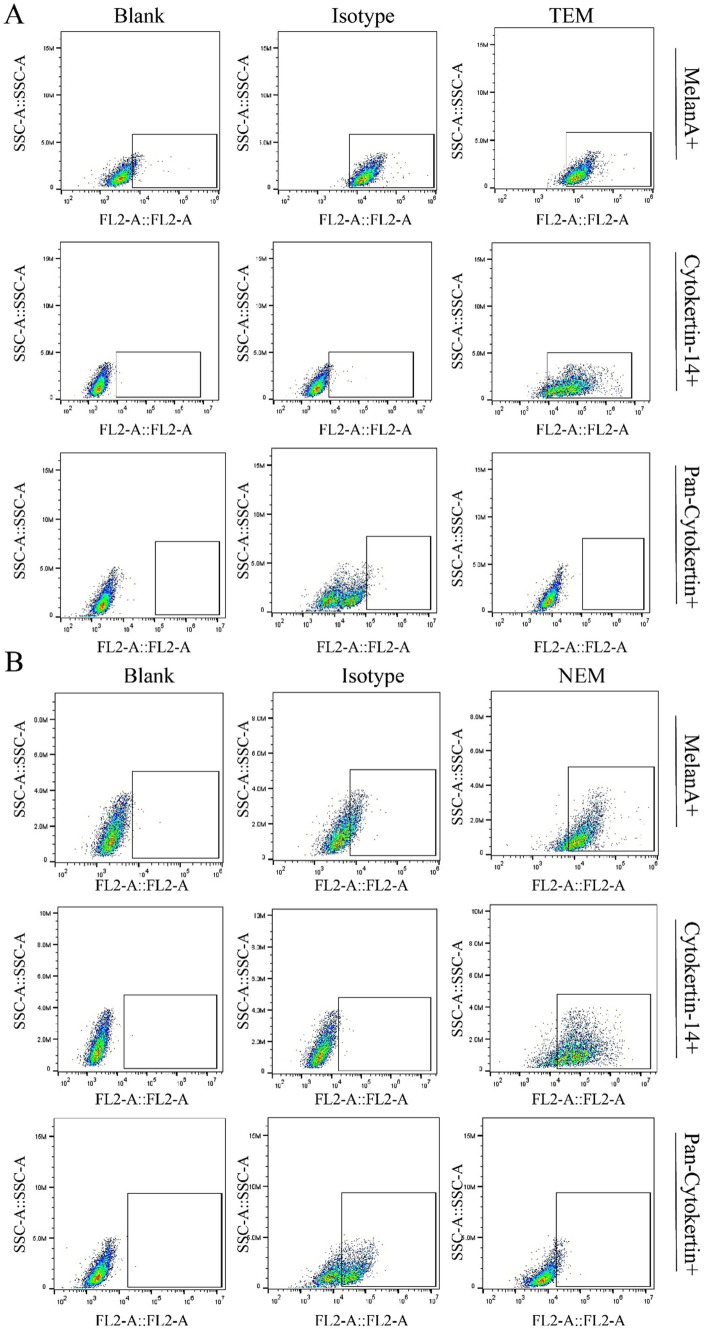
Cell surface markers characterization. The cell markers were identified by flow cytometry. **(A)** Cells obtained via traditional extraction method (TEM) were detected by flow cytometry to evaluate the expressing level of cytokeratin 14, *α*-MelanA and Pan-cytokeratin. **(B)** Cells obtained via new extraction method (NEM) were detected by flow cytometry to evaluate the expressing level of cytokeratin 14, α-MelanA and Pan-cytokeratin.

To assure the safety of the epidermal cells, extracted with our newly developed technology, the cell surface markers were tested by flow cytometry. The key cell markers were detected and compared with cells extracted with clinically by TEM. According to the results, the expression of epidermal cell surface markers obtained by TEM and NEM were basically the same, positively expressing *α*-MelanA and cytokeratin 14, while negative for Pan-cytokeratin (Figure 3). Moreover, MelanA, in conjunction with melanocyte markers, can represent the quantity of melanocytes extracted. Similarly, Cytokeratin 14, Pan-cytokeratin, when paired with keratinocyte markers, signifies the amount of keratinocytes extracted. The results indicated that the proportion of melanocytes extracted by the traditional method is approximately 88.79%, whereas the new method yields about 82.53%. Furthermore, the traditional method extracts keratinocytes at a rate of roughly 92.44%, compared to 84.581% for the new method. The data reveal that, for the same detection volume, the traditional method yields a slightly higher proportion of both melanocytes and keratinocytes than the new method. However, the total number of cells extracted per unit area by the new method is 1.38 times that of the traditional method, with the corresponding total quantities of melanocytes and keratinocytes significantly surpassing those obtained by the traditional approach. Consequently, the new extraction technique demonstrates superior efficacy.

### 3.4 Subcutaneous injection for residence evaluation

**Figure 4:**
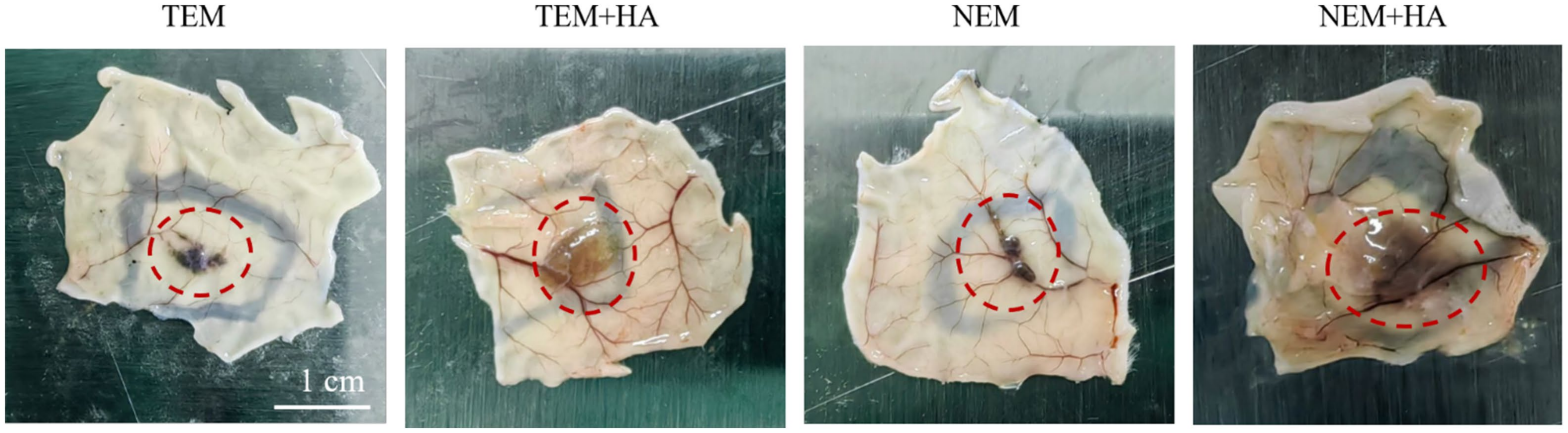
The mouse subcutaneous diagram after 7 days. The red circle indicates cells retention in subcutaneous.

**Figure 5:**
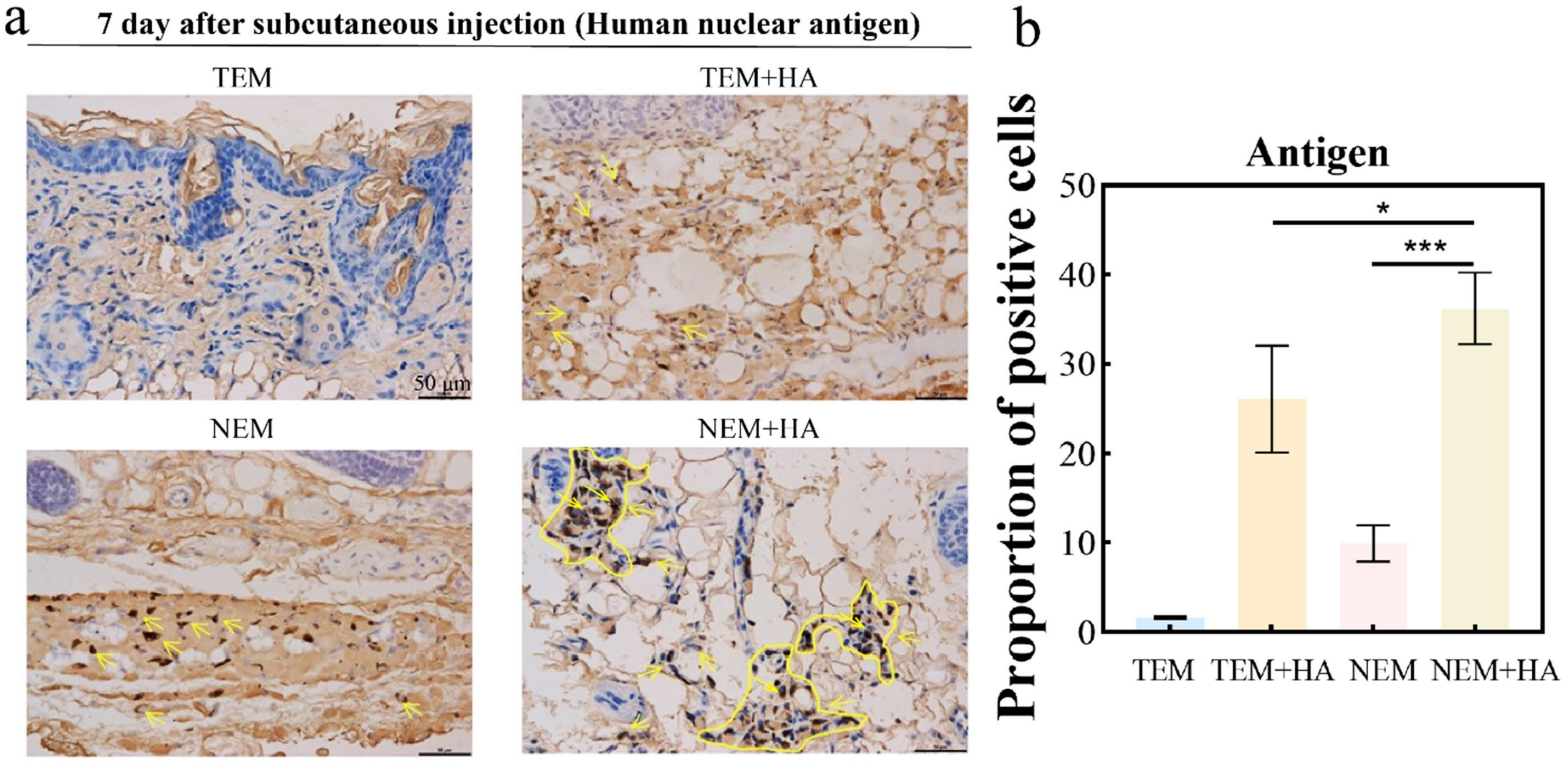
**(a)** Immunohistochemistry of human nuclear antigen; **(b)** Proportion of positive cells about Immunohistochemistry of human nuclear antigen. The yellow arrow indicates cells positively expressing human nuclear antigen (bar: 50 μm) (^*^p < 0.05, ^***^p < 0.001).

**Figure 6:**
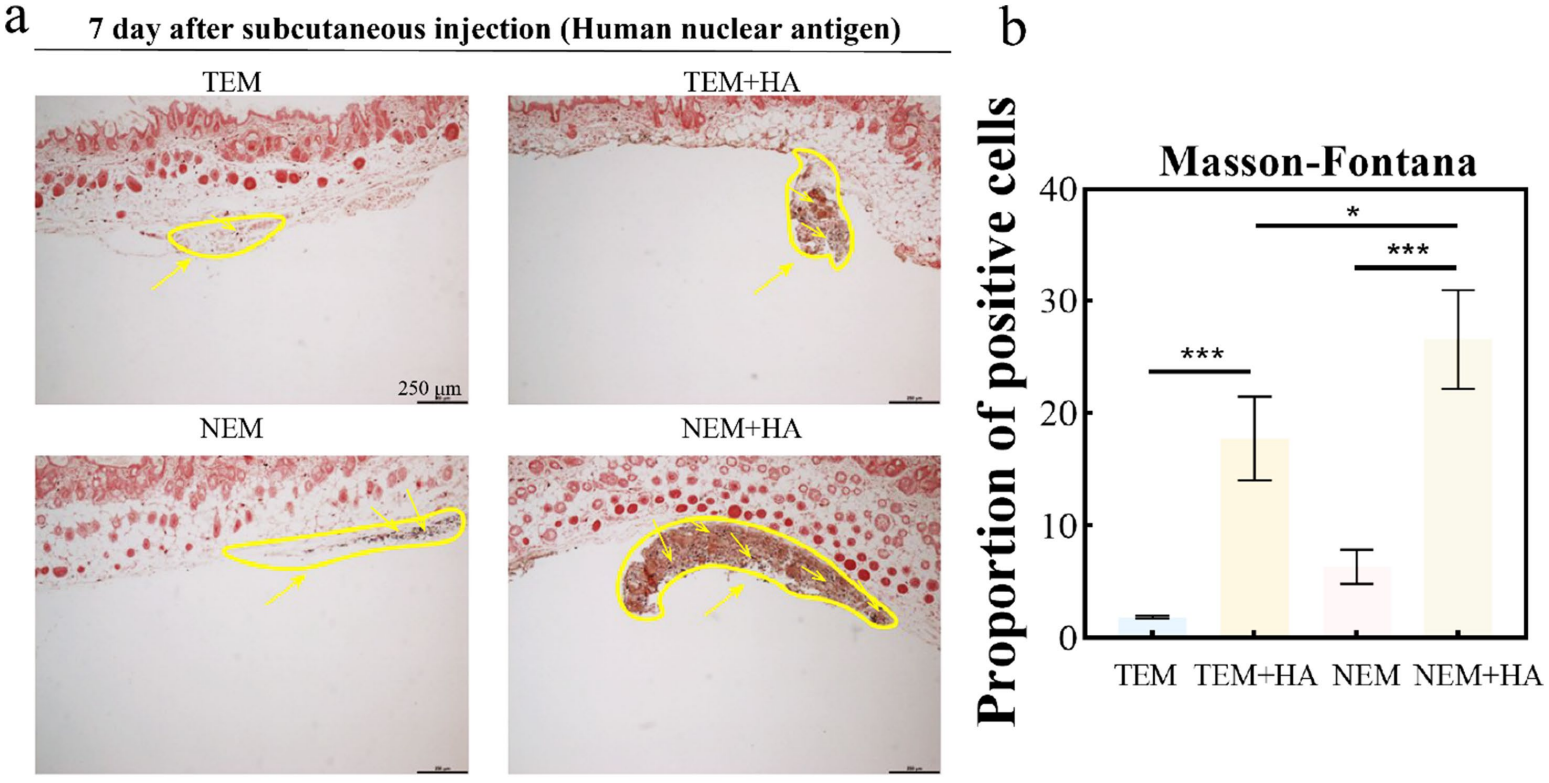
**(a)** Masson-Fontana staining for melanocytes; **(b)** Proportion of positive cells about Masson-Fontana staining for melanocytes. The yellow arrow indicates cells that were positively stained (distribution of melanocytes) (bar: 250 μm) (^*^p < 0.05, ^***^p < 0.001).

To assess whether the crosslinked HA hydrogel could extend the residence time of extracted epidermal cells. The cross-linked HA gel is mixed directly with the extracted cells after sterilization. Therefore, the cell encapsulation efficiency of hydrogel can achieve 100%. The epidermal cell-HA hydrogel suspensions were subcutaneously injected into nude mice. Seven days post-injection, the tissues were harvested, and tissue sections were stained and analyzed. First, human nuclear antigen staining was performed to evaluate the efficacy of the HA hydrogel. The results demonstrated that the HA hydrogel prolonged the retention of cells at the implantation site, as a higher number of cells were observed in the TEM + HA and EEM + HA groups compared to TEM group (Figure 4). During Day 7 inoculation, the inoculation sites of animals in each group were observed. No abnormal skin color changes or subcutaneous nodules were observed at the inoculation sites of animals in the four groups. Furthermore, the NEM + HA group exhibited a greater number of positively stained cells 7 days post-injection about 36.61%. However, the TEM group positively stained cells only exhibited 1.61%. Regardless of the cell extraction method, the introduction of suitable cross-linked HA will increase the cell retention effect (Figure 5). Subsequently, Masson-Fontana staining was conducted to assess the *in vivo* distribution of melanocytes. Similarity, the NEM + HA group exhibited a greater proportion of positively stained cells 25.79%, which is much higher than TEM group (1.86%) (Figure 6). The results revealed that the HA hydrogel effectively retained melanocytes at the implantation site, whereas melanocytes suspended in saline were scarcely detectable at the site. Collectively, these findings suggested that the new extraction method enhanced the in vivo survival rate of cells, while the HA hydrogel significantly prolonged their retention at the implantation site.

## 4 Discussion

Vitiligo is a chronic dermatological condition characterized by localized or generalized depigmentation of the skin, clinically manifested as a reduction in melanin levels (36). The pathogenesis of vitiligo is characterized by a sequence of interrelated pathological events, which can be summarized as follows: (1) Oxidative stress-mediated melanocyte damage, primarily driven by reactive oxygen species (ROS) produced during melanogenesis; (2) Functional decline of melanocytes and melanocyte stem cells; (3) Autoimmune-mediated cytotoxicity targeting melanocytes; (4) Inherent oxidative stress hypersensitivity of melanocytes in vitiligo patients. These mechanisms synergistically contribute to the progressive loss of melanocytes and melanocyte stem cells, resulting in the persistence or expansion of depigmented lesions due to impaired repigmentation capacity. Several treatment modalities are available for vitiligo in clinical practice, among which cell suspension therapy has gained widespread use in recent years, particularly for stable vitiligo cases (37). Conventional cell suspension therapy faces significant limitations, including poor cell viability and suboptimal therapeutic outcomes. To address these challenges, we propose a tissue engineering-based approach that combines biomaterials with cell suspensions for intradermal injection into vitiligo-affected areas. This strategy significantly enhances cellular retention and improves treatment efficacy, representing a promising novel therapeutic paradigm for vitiligo management.

In the context of cell-based therapy, the primary challenges include the prolonged extraction time for epidermal cells, low cell yield, and unstable cell retention at the recipient site. To address these issues, a highly efficient extraction method and protocol was developed. Compared to the traditional technique (0.25% trypsin solution, 4°C, 16 ± 1 h), our approach (0.5% trypsin solution, 37°C, 2 ± 0.5 h) offers two significant advantages: (1) the extraction time for the epidermal cell suspension was significantly reduced to approximately 2 h without compromising cell viability or functionality; and (2) crosslinked sodium hyaluronate was employed as a carrier for the cell suspension, enhancing cell retention and prolonging its duration at the implantation site.

To assess the biosafety of our approach, comprehensive cell surface marker characterization was performed (Figure 3). Comparative analysis revealed identical expression profiles of epidermal cell markers between cells isolated via conventional and novel extraction methods. Specifically, positive expression of *α*-MelanA and cytokeratin 14, coupled with negative pan-cytokeratin staining, confirmed maintenance of normal cellular phenotypes. To evaluate therapeutic efficacy, we conducted *in vivo* studies using a nude mouse model. Masson-Fontana melanin staining and immunohistochemical analysis (Figures 5, 6) demonstrated that HA hydrogel-mediated delivery significantly enhanced cellular persistence at the implantation site. Quantitative assessment revealed substantially greater cell retention in HA hydrogel-treated groups compared to controls (*p* < 0.01), confirming the biomaterial’s capacity to improve cellular engraftment.

Our findings demonstrate the development of a promising therapeutic approach for stable vitiligo. However, clinical validation through further human studies remains imperative. As a next step, we plan to initiate a hospital-based pilot clinical trial with eligible participants. To enhance clinical applicability, particularly for patients with extensive lesions, scaling up cellular expansion prior to transplantation will be critical. Current *in vitro* expansion protocols predominantly utilize animal serum-containing media, which presents non-negligible biosafety risks including potential contamination with pathogens (bacteria, viruses, or prions) that could compromise patient safety upon administration. To address these limitations, there is an urgent need to develop serum-free culture media specifically optimized for the in vitro expansion of both epidermal keratinocytes and melanocytes. This advancement would provide a safer, more standardized, and clinically translatable approach for cell-based vitiligo therapies.

## 5 Conclusion

In summary, this study developed an injectable hyaluronic acid (HA)-based cell delivery system for the treatment of stable vitiligo. First, we optimized the cell extraction protocol to safely obtain a sufficient quantity of epidermal cells within approximately 2 h. Second, the use of crosslinked hyaluronic acid hydrogel as a cell delivery system significantly enhanced the retention of transplanted cells, including a substantial population of melanocytes. Together, these advancements provide a promising cell-based therapeutic strategy for the treatment of stable vitiligo.

## Data Availability

The original contributions presented in the study are included in the article/Supplementary material, further inquiries can be directed to the corresponding authors.
