# Supplementary material for: Injectable crosslinked HA hydrogel: a promising carrier for cell transplantation to treat stable vitiligo
Source: Front Med (Lausanne). 2025 May 12;12:1583271. doi: 10.3389/fmed.2025.1583271 (PMC12104057; doi:10.3389/fmed.2025.1583271)
Supplement: Supplementary file 1 [file Supplementary_file_1.docx]

Injectable crosslinked HA hydrogel: a promising carrier for cell transplation to treat stable vitiligo

Qianren Zheng^1†^, Jie Chen^2†^, Yixun Huang^3†^, Weikai Chen^3^, Dandan Cheng^4^ Qianqian Jia^1^, Meiqin Zhu^1^, Youguo Liao^7*^, Qiulin He^1,6*^, Shunli Wu^5, 1*^

^1^Hangzhou Singclean Medical Products Co., Ltd, Hangzhou, China.

^2^Jiaxing Vocational Technical College, Department of Student Affairs, Jiaxing, Zhejiang, China

^3^Department of Orthopedics, The Second Affiliated Hospital and Yuying Children’s Hospital of Wenzhou Medical University, Wenzhou, Zhejiang 325003, China.

^4^Department of Gynecological Oncology, Wenzhou Central Hospital, Wenzhou, Zhejiang 325000, China.

^5^College of Marine Life Sciences, Ocean University of China, Qingdao, Shandong, China

^6^Department of Macromolecular Science, Fudan University, Shanghai, China

^7^Department of Burns and Wound Care Center, Second Affiliated Hospital, College of Medicine, Zhejiang University, Hangzhou, 310009, China


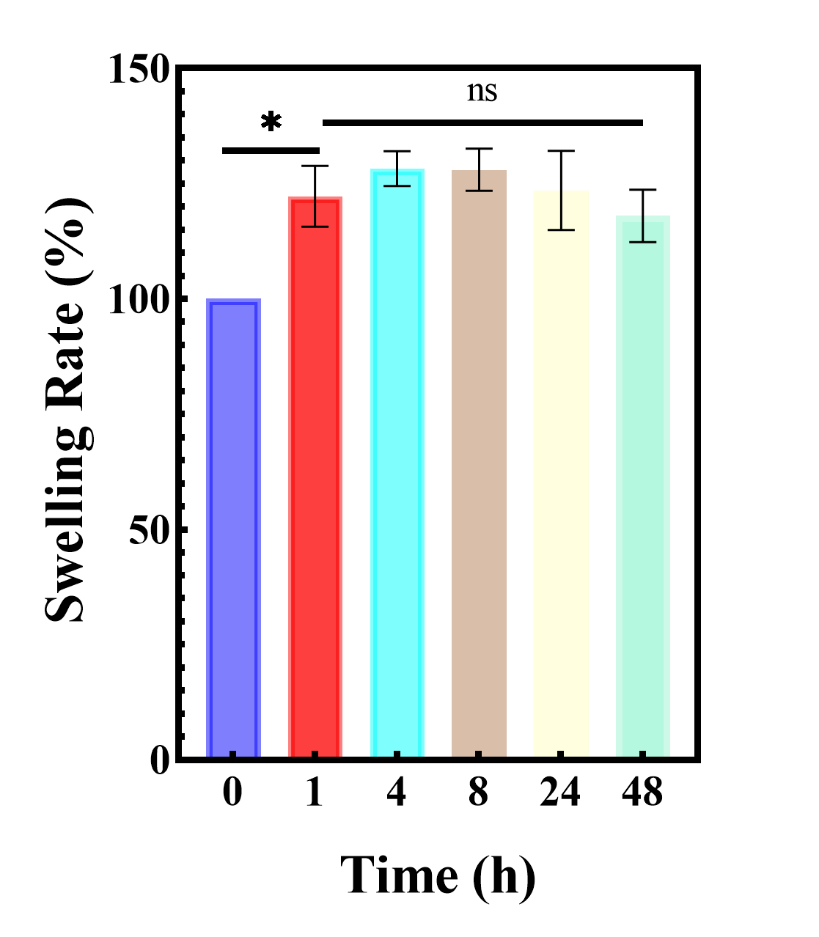


Figure S1. The balance swelling rate of crosslink HA.


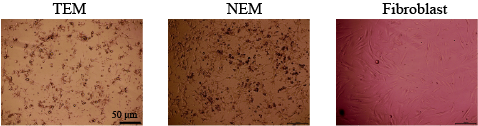


Figure S2. Extract the morphology of the cells. Bar: 50 μm


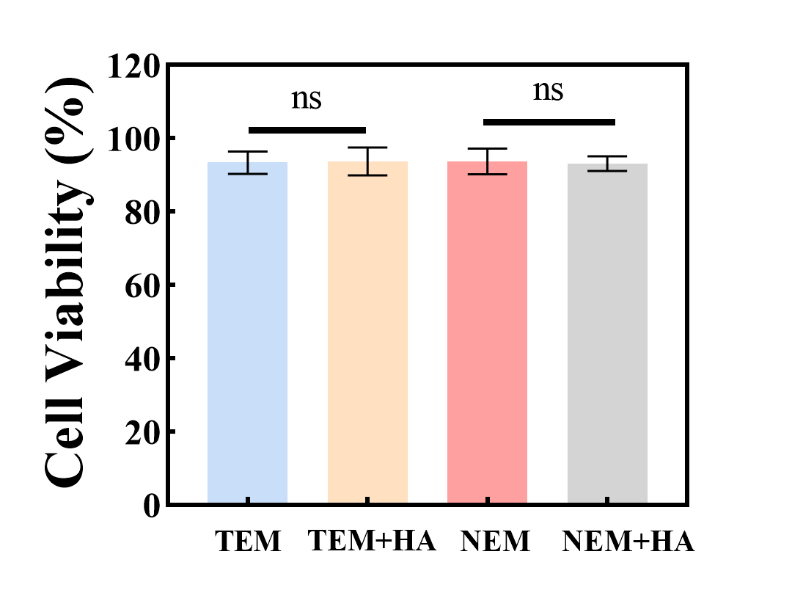


Figure S3. The cell viability of TEM and NEM with crosslinked HA hydrogel.
